# Gram stain-guided antibiotic choice: a GRACEful method to safely restrict overuse of broad-spectrum antibiotic agents

**DOI:** 10.1186/s13054-018-2270-z

**Published:** 2018-12-14

**Authors:** Jumpei Yoshimura, Kazuma Yamakawa, Takahiro Kinoshita, Satoshi Fujimi

**Affiliations:** Division of Trauma and Surgical Critical Care, Osaka General Medical Center, 3-1-56 Bandai-Higashi, Sumiyoshi, Osaka, 558-8558 Japan

The rapid pandemic spread of multidrug-resistant (MDR) pathogens and the paucity of new, effective antibiotics are placing patients’ safety at risk worldwide [1]. The World Health Organization (WHO) adopted a global action plan on antimicrobial resistance, emphasising the need to optimise the use of antibiotic agents [2]. We recently reported in *Critical Care* the effectiveness of Gram stain results to reduce the use of broad-spectrum antibiotics [3]. Here, we would like to report the prospective validation of the usefulness of Gram staining for antibiotic choice.

We conducted a prospective observational study from July 2016 to June 2017. Patients diagnosed as having ventilator-associated pneumonia (VAP), defined by a modified clinical pulmonary infection score ≥ 5, were enrolled and treated according to a Gram stain-guided antibiotic choice algorithm (Fig. 1). The primary outcome was clinical response of VAP (Additional file 1: Table S1).

**Fig. 1:**
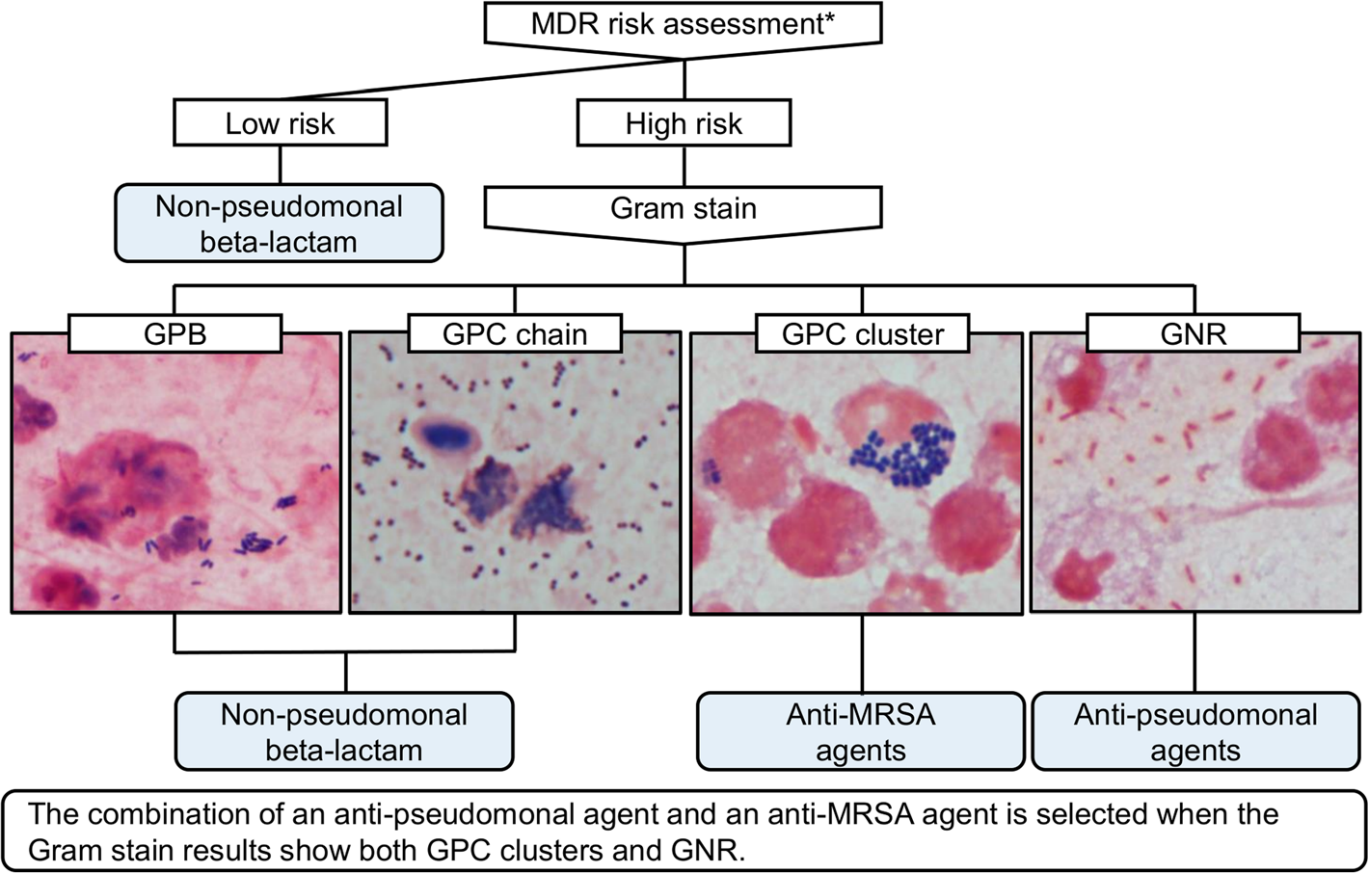
Algorithm of Gram stain-guided antibiotic choice. *Patients with any of the following conditions considered at high risk: antimicrobial therapy in preceding 90 days, hospital stay of 96 h or longer, chronic dialysis, immunosuppressive disease or therapy, nursing home admission, septic shock at time of ventilator-associated pneumonia or colonising MDR pathogens. GPB Gram-positive bacilli, GPC Gram-positive cocci, GNR Gram-negative rods, MDR multidrug-resistant, MRSA methicillin-resistant *Staphylococcus aureus*

Nineteen patients with a median age of 65 (49–81) years were enrolled during the study period. Clinical risk factors for MDR pathogens were present in 13 (68.4%) of the VAP patients. Pathogens isolated from endotracheal aspirates are presented in Additional file 2: Table S2. The primary outcome of the clinical response rate of VAP was 68.4%, which was comparable to previous trials using broad-spectrum antibiotics in similar clinical settings (Additional file 3: Table S3). Treatment failure occurred in six patients: antibiotic therapies were continued for more than 14 days in three patients, and pneumonia relapsed within 7 days after the end of therapy in the other three patients. The administered antibiotics did not cover pathogens isolated from an endotracheal aspirate in one patient (5.3%). The algorithm proposed narrower-spectrum antibiotics in 15 patients (78.9%) than those proposed by the 2016 Infectious Diseases Society of America–American Thoracic Society VAP guidelines [4] (Fig. 2). We restricted the use of anti-methicillin-resistant *Staphylococcus aureus* agents and anti-pseudomonal agents in 12 and 10 patients, respectively.

**Fig. 2:**
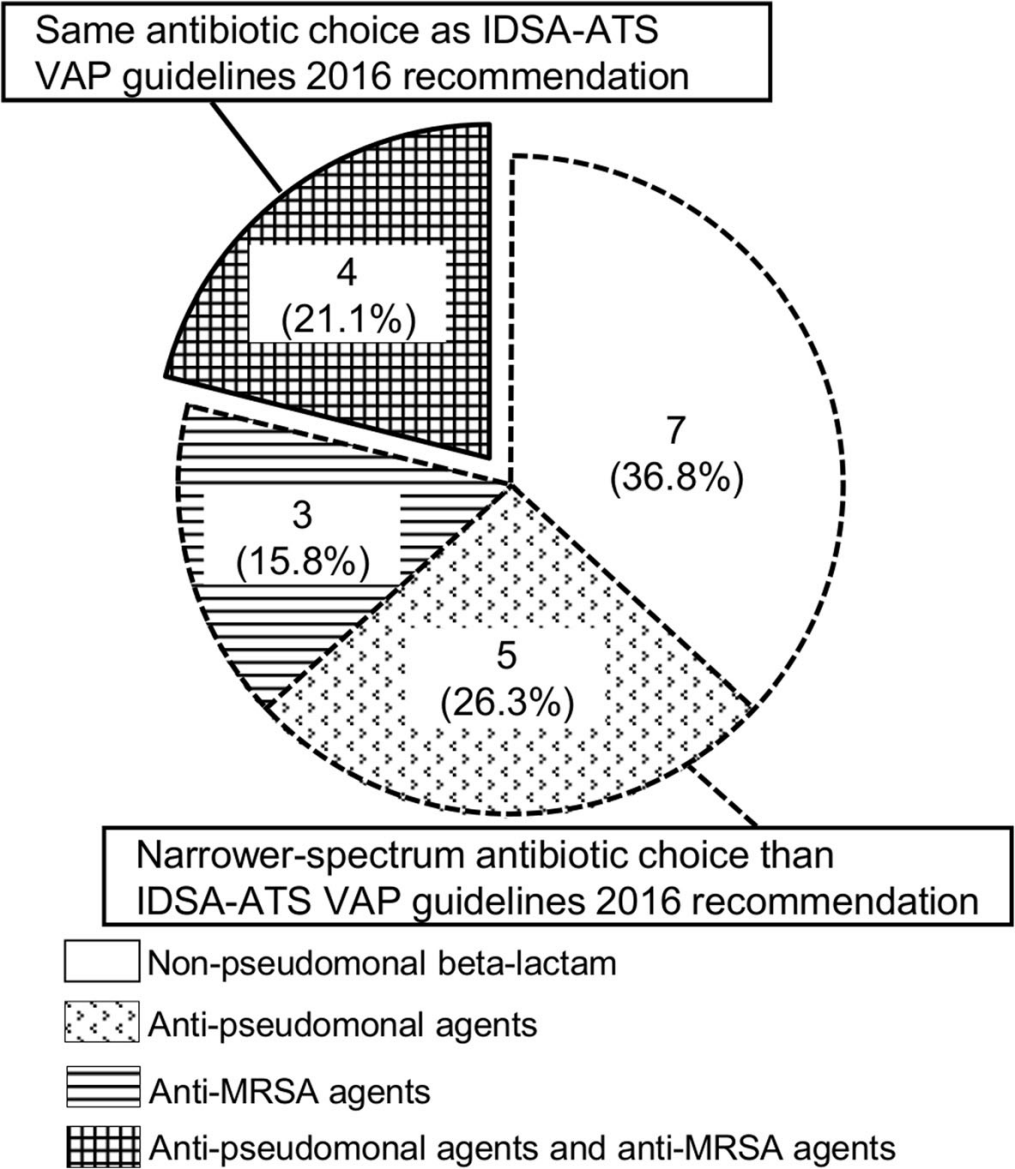
Details of administered antibiotic agents. IDSA-ATS Infectious Diseases Society of America–American Thoracic Society, MRSA methicillin-resistant *Staphylococcus aureus*, VAP ventilator-associated pneumonia

Our Gram stain-guided antibiotic choice algorithm was shown not only to safely guide appropriate initial antibiotic therapy but also to properly cure VAP. On the basis of the promising results of this study, we are conducting a multicentre, randomised, non-inferiority trial (GRam stain-guided Antibiotics ChoicE for Ventilator-Associated Pneumonia (GRACE-VAP)) to compare our Gram stain-guided treatment with guidelines-based treatment for patients with VAP (ClinicalTrials.gov NCT03506113, registered on 29 March 2018) [5]. Because Gram staining is an inexpensive examination and is easy to perform worldwide, including in developing countries, it could be a GRACEful method to optimise the use of antibiotics safely throughout the world.

## Additional files

Additional file 1: **Table S1.** Definition of clinical response of ventilator-associated pneumonia. (DOCX 21 kb)

Additional file 2: **Table S2.** Pathogens associated with ventilator-associated pneumonia. MRSA: methicillin-resistant Staphylococcus aureus. (DOCX 22 kb)

Additional file 3: **Table S3.** Clinical response in the present study and previous studies. VAP: ventilator-associated pneumonia; HAP: hospital-acquired pneumonia. (DOCX 22 kb)

## Abbreviations

MDR: Multidrug resistant
VAP: Ventilator-associated pneumonia
WHO: World Health Organization
