# Supplementary material for: Gram stain-guided antibiotic choice: a GRACEful method to safely restrict overuse of broad-spectrum antibiotic agents
Source: Crit Care. 2018 Dec 14;22:338. doi: 10.1186/s13054-018-2270-z (PMC6295032; doi:10.1186/s13054-018-2270-z)
Supplement: Supplementary file 1 — Table S1. Definition of clinical response of ventilator-associated pneumonia. (DOCX 21 kb) [file 13054_2018_2270_MOESM1_ESM.docx]

| **Table S1. Definition of the clinical response of ventilator-associated pneumonia** |
| --- |
| Fulfilment of all four of the following components: |
| 1) Completion of antibiotic therapy within 14 days |
| 2) Improvement or lack of progression of baseline radiographic findings at the end of therapy |
| 3) Resolution of signs of pneumonia 7 days after the end of therapy |
| 4) Resolution of symptoms of pneumonia 7 days after the end of therapy |
|  |
