# Supplementary material for: Gram stain-guided antibiotic choice: a GRACEful method to safely restrict overuse of broad-spectrum antibiotic agents
Source: Crit Care. 2018 Dec 14;22:338. doi: 10.1186/s13054-018-2270-z (PMC6295032; doi:10.1186/s13054-018-2270-z)
Supplement: Supplementary file 2 — Table S2. Pathogens associated with ventilator-associated pneumonia. MRSA: methicillin-resistant Staphylococcus aureus. (DOCX 22 kb) [file 13054_2018_2270_MOESM2_ESM.docx]

| **Table S2. Pathogens associated with ventilator-associated pneumonia** | |  |
| --- | --- | --- |
| Pathogen | Number of patients (%) | |
| Gram-positive bacteria | 17 (89.5%) | |
| *Staphylococcus aureus* | 7 (36.8%) | |
| MRSA | 3 (15.8%) | |
| *Streptococcus pneumoniae* | 1 (5.3%) | |
| Other streptococci | 9 (47.4) | |
| Gram-negative bacteria | 11 (57.9%) | |
| *Klebsiella* spp*.* | 3 (15.8%) | |
| *Enterobacter* spp*.* | 3 (15.8%) | |
| *Pseudomonas aeruginosa* | 2 (10.5%) | |
| *Acinetobacter baumannii* | 1 (5.3%) | |
| *Citrobacter* spp*.* | 1 (5.3%) | |
| *Escherichia coli* | 1 (5.3%) | |
| MRSA: methicillin-resistant *Staphylococcus aureus* |  | |
