# Supplementary material for: Gram stain-guided antibiotic choice: a GRACEful method to safely restrict overuse of broad-spectrum antibiotic agents
Source: Crit Care. 2018 Dec 14;22:338. doi: 10.1186/s13054-018-2270-z (PMC6295032; doi:10.1186/s13054-018-2270-z)
Supplement: Supplementary file 3 — Table S3. Clinical response in the present study and previous studies. VAP: ventilator-associated pneumonia; HAP: hospital-acquired pneumonia. (DOCX 22 kb) [file 13054_2018_2270_MOESM3_ESM.docx]

| **Table S3. Clinical response in the present study and previous studies** | | | | | | |
| --- | --- | --- | --- | --- | --- | --- |
|  | | | Study drugs  (Number of patients) | | Clinical response rates | |
| Source | Population | Intervention | | Control | Intervention | Control |
| Chastre *et al.* | VAP | Doripenem  with/without anti-MRSA agents  (n=249) | | Imipenem  with/without anti-MRSA agents  (n=252) | 59.0% | 57.8% |
| Rubinstein *et al* | HAP | Telavancin  with/without anti-pseudomonal agents  (n=749) | | Vancomycin  with/without anti-pseudomonal agents  (n=754) | 58.9% | 59.5% |
| Kollef *et al* | VAP | Doripenem  with/without anti-MRSA agents  (n=115) | | Imipenem-cilastatin  with/without anti-MRSA agents  (n=112) | 45.6% | 56.8% |
| Award *et al* | HAP | Ceftobiprole  with/without fluoroquinolone or aminoglycoside  (n=391) | | Ceftazidime plus linezolid  with/without fluoroquinolone or aminoglycoside  (n=390) | 49.9% | 52.8% |
| Present study | VAP | Gram stain-based antibiotic therapy  (n=19) | | - | 68.4% | - |
| VAP: ventilator-associated pneumonia; HAP: hospital-acquired pneumonia | | | | | | |
